# Reducing Waste in 3D Printing Using a Neural Network Based on an Own Elbow Exoskeleton

**DOI:** 10.3390/ma14175074

**Published:** 2021-09-04

**Authors:** Izabela Rojek, Dariusz Mikołajewski, Jakub Kopowski, Piotr Kotlarz, Maciej Piechowiak, Ewa Dostatni

**Affiliations:** 1 Institute of Computer Science, Kazimierz Wielki University, Chodkiewicza 30, 85-064 Bydgoszcz, Poland; dmikolaj@ukw.edu.pl (D.M.); kopowski@ukw.edu.pl (J.K.); piotrk@ukw.edu.pl (P.K.); mpiech@ukw.edu.pl (M.P.); 2 Institute of Materials Technology, Faculty of Mechanical Engineering, Poznan University of Technology, pl. M. Skłodowskiej-Curie 5, 60-965 Poznan, Poland; ewa.dostatni@put.poznan.pl

**Keywords:** neural network, 3D printing, reduction of waste, elbow exoskeleton

## Abstract

Traditional rehabilitation systems are evolving into advanced systems that enhance and improve rehabilitation techniques and physical exercise. The reliable assessment and robotic support of the upper limb joints provided by the presented elbow exoskeleton are important clinical goals in early rehabilitation after stroke and other neurological disorders. This allows for not only the support of activities of daily living, but also prevention of the progression neuromuscular pathology through proactive physiotherapy toward functional recovery. The prices of plastics are rising very quickly, as is their consumption, so it makes sense to optimize three dimensional (3D) printing procedures through, for example, improved artificial intelligence-based (AI-based) design or injection simulation, which reduces the use of filament, saves material, reduces waste, and reduces environmental impact. The time and cost savings will not reduce the high quality of the products and can provide a competitive advantage, especially in the case of thinly designed mass products. AI-based optimization allows for one free print after every 6.67 prints (i.e., from materials that were previously wasted).

## 1. Introduction

The elbow joint is the most complex human joint and is crucial from the point of view of upper limb mobility for activities of daily life, study, work, and leisure (including sports). The elbow joint is affected by a number of specific conditions (tennis elbow, degeneration, ossification) and any deficit in this area significantly affects the subjective quality of life, thus functional deficits of the elbow joint require a personalized solution to partially relieve and support the movement sequences characteristic of this joint. The prevalence of function deficit, weakness, or fatigability of upper limbs is not geographically subdivided. There are 400–800,000 strokes per year in Poland, this being the most common cause of such deficits, for which there are no alternatives except the one proposed here. In addition, a significant group of such patients are elderly people (6–10 million Poles) with similar negative deterioration of upper limb function due to neurodegenerative changes in the aging process [1]. Three modes of rehabilitation are used at different stages after a stroke:

- In the acute phase: external force-based control;
- Middle phase of recovery: supportive force-based rehabilitation; and
- Last phase: resistance-based rehabilitation [2].

An elbow joint exoskeleton is a technical device that extends and improves selected skills of the user. From a medical perspective, an elbow exoskeleton can serve as a multifunctional medical device, improving the patient’s capabilities in a manner that approximates the natural use of upper limb function (one or both). In addition, exoskeletons are considered to be more effective than traditional therapist support and care using rehabilitation devices for the following reasons: (1) the exoskeleton for the upper limb is individually tailored to the type and degree of deficit and anthropometric parameters of the patient (thanks to the use of 3D scanning and 3D printing); (2) the use of the exoskeleton is close to the natural functioning of the patient, so the patient does not have to learn the function of the upper limb (fingers of the hand, wrist) from the beginning; (3) use of the exoskeleton lasts (in some cases) 24 h a day, seven days a week, and is not limited only to the hours spent with a therapist, which significantly extends the duration of the active therapeutic effect of the device and increases the effectiveness of therapy; (4) adjustment of the device is relatively quick; and (5) the device is relatively simple and inexpensive, and so is adapted to the realities of the market. This paper proposes a new functionality: a passive exoskeleton (support based on elastic devices) and an active exoskeleton (support based on actuators) for people with function deficit and/or muscle strength weakness in the elbow joint area. The solution develops the concept of personalized therapy. 3D scanning makes it possible to record the characteristics of the upper limb structure as digital files. The combination of 3D scanning technology and 3D printing in the form of reverse engineering provides the capability to relatively inexpensively create (with adaptation to a specific user) a digital design of the exoskeleton with a complex internal and external structure that is based on the physical equivalent (including anatomical). 3D printing makes it possible to create an exoskeleton from digital files, with parameters selected individually for each patient (dimensions, strength, flexibility, weight, support strength, etc.).

The solution proposes a new quality:

1. Quality advantages:
  - Individual fit and manufacturing by 3D printing;
  - Supporting the function of the whole upper limb (using more than one module) or its selected parts (elbow joint);
  - Use in children and adults;
  - Immediate improvement of functions (reaching, interaction with objects); and
  - Shaping improvement of function over a longer period of time.
2. Technological advantages:
  - Possibility of daily use in the home environment; and
  - Gradual adaptation (in the future by replacing components) to changes in health status.
3. Environmental advantages—solutions that change with the patient (with adjustable or replaceable components, no need to replace the entire device).
4. Cost advantages:
  - Domestic production, lower costs; and
  - On-site availability (including exams and scans).

Similar solutions have been developed in several countries (e.g., in the USA, and in Singapore [3]) as prototypes, but to our knowledge, they have not entered into mass production. A prototype of a stationary elbow exoskeleton has been developed by a Polish–Italian team with the participation of the Collegium Medicum Nicolaus Copernicus University (CM UMK) and University of Science and Technology (UTP) [4], but despite its technical sophistication, it can serve as a stationary elbow joint rehabilitation tool on the rehabilitation center’s equipment, but it cannot serve as a wearable individual elbow joint support that is fully mobile with the patient. For the aforementioned reasons, this solution is technically and purposefully very different from the 3D-printed mobile elbow exoskeleton solution proposed in this paper.

The result can be used in the medical industry (biomedical engineering, rehabilitation supplies). The most important users are patients with elbow joint function deficits and/or upper limb strength weakness, and their caregivers. Doctors and physiotherapists are an important group of stakeholders, but the ones who will benefit as a result of the implementation of this innovation are their patients (faster and cheaper diagnostics, reduced queues to specialists, the possibility of rehabilitation in a new, more patient- and family-friendly form).

Spasticity assessment and functional rehabilitation based on the precision and accuracy of the elbow exoskeleton combined with electromyography (EMG) recording and the physiotherapist’s experience are complementary, and through synergy, maximize the benefits of both practices [5,6]. The aforementioned accuracy of the upper limb activation model, according to a study by Jiang et al., for:

- Convolutional neural network (CNN) in recognizing movement patterns from surface EMG was 97.57% for normal-speed movements and 97.07% for fast-speed movements [7];
- Cross-subject CNN in motion pattern recognition was 79.64% [7]; and
- Cross-device CNN in motion pattern recognition was 88.93% for normal-speed movements and 80.87% for mixed-speed movements [7].

Traditional rehabilitation systems are evolving into advanced systems based on exoskeletons and virtual reality (VR) environments that enhance and improve rehabilitation techniques and physical exercise. Robotic exoskeletons aid in motor re-education. Chen et al. showed a performance suitable for robotic therapy applications for an elbow joint exoskeleton:

- Torque errors of less than 0.19 Nm [8];
- Torque rise time of 0.1 s [8];
- Torque control bandwidth of 3.7 Hz [8];
- Impedance less than 0.03 Nm/deg at 1 Hz [8]; and
- Maximum stiffness of 0.45 Nm/deg [8].

Despite the efforts of researchers, the solutions developed so far have stopped at the prototype stage [9,10,11,12,13,14,15,16].

### Optimization of Solutions

The idea of optimizing 3D printing and control systems is central to the development of this group of technologies by relying on new printing technologies, acquisition of control signals, their classification and interpretation, novel mechanical properties of materials (including ease of disinfection), and automation of their use in 3D printing (including multi-material printing) [17,18,19,20,21].

In our case, the research work focused on the design of a lightweight and ergonomic 3D printed elbow exoskeleton designed to support the function and rehabilitation of the upper limb, and capable of generating diverse training scenarios. For the aforementioned reasons, the kinematics and material properties of the aforementioned structure are subject to optimization in terms of the prototype design itself, the motility of the device from the point of view of the patient, their family/caregivers, and the therapist, and the manufacturing of the exoskeleton as a product. It becomes crucial to integrate and match the 3D-printed exoskeleton with passive gravity-compensating mechanisms and with potential active rotating elements, hence the high requirements in terms of 3D printing and materials for this. Fused deposition modeling (FDM) is one of the most widespread, versatile, and accessible 3D printing techniques. The material (polymer in the form of a filament) is melted and extruded onto the surface layer by layer, and the molten material of the new layer adheres to the previous layers. With regard to the production of exoskeletons, PLA filaments are characterized by higher adhesion strength, but the set of printing parameters (see Table 1) has remained unexplored thus far.

The choice of material and 3D printing technology for specific components must be optimized, especially as the number of materials and technologies available on the market is increasing, hence the need to formulate requirements and selection criteria. In the selection process, we investigated different materials to enable the construction and quasi-series production of a personalized exoskeleton, and in the course of the research, the proposed material proved to be good and cheap.

Artificial neural networks (ANN) allow the exploration, modeling, and prediction of relationships between datasets: input and output, reflecting non-linear relationships between them that are difficult to capture using traditional analysis methods. Functionally speaking:

- Connections between neurons in successive ANN layers (input, hidden, output) are strengthened by similarities in the measured datasets;
- Complex, incomplete or noisy datasets are not an obstacle;
- Input data aggregation and hidden layer transfer function are used to calculate the response (estimate), thanks to a learning process that is more accurate, sensitive and specific than traditional statistical procedures;
- The networks learn as new data becomes available; and
- Inference and estimation of latent relationships between large sets of features allow optimal and innovative ways to solve even complex technological problems to be found.

Multiparametric optimization problems are difficult, complex, and time consuming because:

- They often require optimization methods that minimize or maximize certain objective functions;
- Sometimes, the problems that have to be optimized are not linear or polynomial;
- Some problems cannot be solved exactly and must be approximated; and
- Some optimization problems require the use of heuristics, since no other method can provide an efficient solution in a reasonable time.

Some algorithms linearize constraints and objective functions at a specific point in space, using derivatives and partial derivatives in some cases, while in other cases, evolutionary algorithms are used to approximate the solution [22].

Automatic or semi-automatic artificial intelligence analysis of data from the manufacturing process in real or near real-time is a practical implementation of technical and environmental control at each stage of production within the paradigm of Industry 4.0. Comprehensive collection of process parameters and construction of virtual twins of the production line and the product at various stages of its creation make it possible to maintain an individual approach to 3D printing of the object while standardizing the procedures of its design, manufacturing, and monitoring as well as analysis and control of its life cycle.

Dynamic models providing the theoretical basis, description of physical phenomena, and mechanisms of their interaction within the 3D printing process considering dynamic self-consistency, resonant response, and damping have already been developed [23]. The residual porosity of the part affects the mechanical properties of PLA reinforced with short polylactic acid fibers printed by multiaxial material extrusion (MAMEX). This results in the need to optimize the 3D printing process to avoid weakening of the print stiffness by a lack of anisotropic behavior and mechanical optimization (i.e., fiber alignment with respect to internal stresses) [24]. The optimization of the additive printing process from polymers, composites, geopolymers, and new materials is also influenced by the characteristics of the new printers including the ability to print from the aforementioned materials for various applications [25].

In the course of research, individual parts of the exoskeleton prototypes and samples of materials for 3D printing of the exoskeleton were subjected to strength tests: tensile, shear, and torsion. This helps to ensure the required error margins and the associated patient safety. This is a major challenge as the exoskeleton is personalized (i.e., tailored to the size and strength of the patient’s upper limb, as well as the patient’s type and degree of deficit, i.e., the support required by the exoskeleton). For the above-mentioned reasons, we tested standardized elements, and the target elements of the exoskeleton may be of different sizes, however, with recalculation of strength test results and testing of individual finished elements.

The aim of the study was to investigate whether AI-based optimization can improve the environmental friendliness of the elbow exoskeleton without compromising its quality.

## 2. Materials and Methods

**Figure 1:**
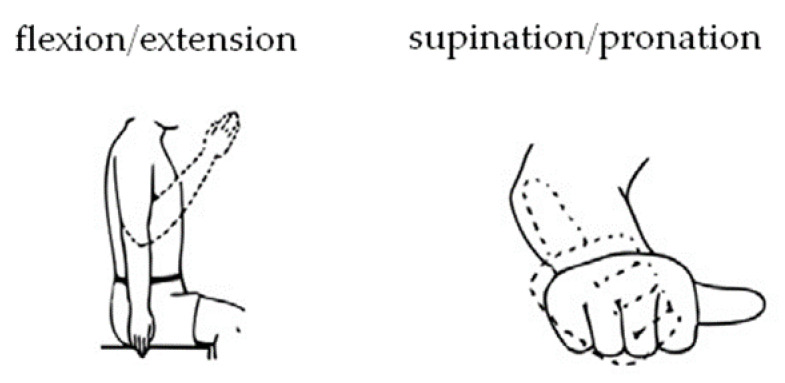
Supported elbow movements [26].

**Figure 2:**
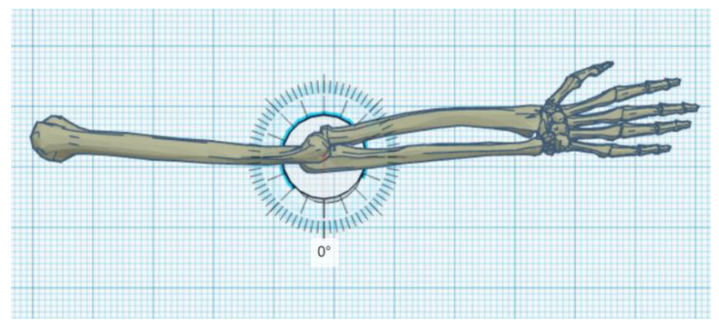
Straighten elbow with marked 120° angle of rotation.

**Figure 3:**
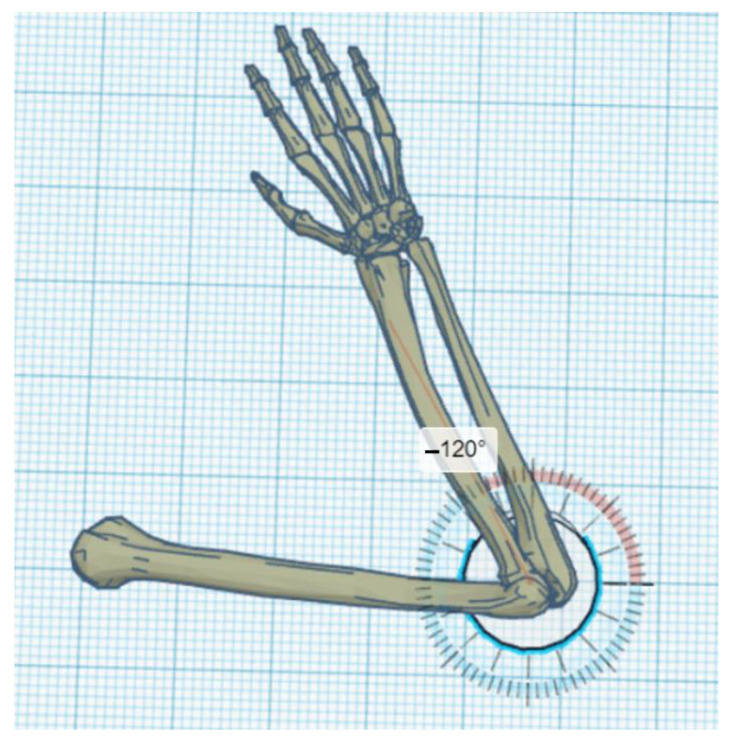
Maximum flexion of the elbow with a marked angle of rotation of 120°.

The exoskeleton should provide a full range of motion (i.e., from a straight elbow (0°) to full flexion of about 120°–130°) (Figure 1, Figure 2 and Figure 3).

It should be noted that these values can be different, for example, for acrobats and gymnasts whose stretching exercises can increase the minimum and maximum flexion of the elbow and other joints. For bodybuilders, on the other hand, the increase in muscle tissue limits the full range of motion of the joint, especially the elbow, and thus impairs the movements of such people and prevents the proper performance of various activities.

The project used two concepts: external (concept 1) and internal (concept 2).

As in mechanics including in the case of a human body, models are built and used. A mechanical model of a human being is understood as a mechanical system with concentrated or distributed masses, whose mechanical impedance or transmittance is in accordance, according to a certain criterion, with the found impedance or transmittance of a human body. From the point of view of mechanics, the human body can be regarded as a continuous system. Such a system can be simplified. We will analyze the following concepts/types of model:

- Conceptual model—represents some logical proposal for organizing the essential features of a process (by this, we mean a high degree of simplification of the model relative to the original system);
- Physical model—the system to be studied and the phenomena occurring in it. It gives interpretations to the features of the process, consistent with the physical nature of the process (a physical model is understood as a faithful replica of a considered process or system on a reduced scale);
- Mathematical model—an analytical description of the studied phenomena covered by the physical model. It represents model features in the form of mathematical relations (a mathematical model is understood as a set of mathematical relations describing a system);
- Structural models—the internal organization of the model is similar to the internal organization of the system under study, in fact, there is a similarity and correspondence between the elements of the model and the elements of the system; and
- Functional models—their construction does not go into the internal structure of the system, and only fulfil the condition that the input signals result in output signals sufficiently close to the output signals of the object.

Furthermore, we classified models according to the nature of the input values:

- Discrete model takes only specified values; and
- Continuous model takes arbitrary values within a specified range.

A model is a certain simplification, an idealization of the modeled system. The correspondence between the model and the system should be high enough so that any conclusions concerning the system based on simulation studies of the model can be considered true.

It is not possible to give a general prescription for building models. However, for models with a specific purpose, some methodological guidance is possible.

A model should be evaluated in terms not of what it is supposed to forecast, but how well it forecasts what it is supposed to forecast. Therefore, a simple model that makes a specific claim may be “better” than a complex model.

A simple model can be beneficial in the following cases:

- If it makes a more specific thesis; and
- If its accuracy in predicting the information it is used to determine is greater than that of a complex model.

It is also necessary to define what the model represents and what it does not represent—detailed criteria. This may relate to:

- The form of the model;
- Model parameters;
- Conditions under which the model can be applied;
- Applications of the model; and
- Model accuracy.

The current economy, especially in the area of plastics, is faced with an excess of waste, hence, for example, the recent introduction in the EU of a ban on the use of disposable cutlery made from this group of materials. Environmental friendliness is becoming an important criterion in production—and a forward-looking technology such as 3D printing cannot avoid it. 3D printing of the exoskeleton, although clinically, economically, and socially important, must also fit into these requirements. Hence, the optimization of 3D printing to reduce waste.

The use of artificial neural networks (ANNs) to approximate the objective function in optimization problems is used quite frequently including as an intermediate solution to allow other techniques to be applied at later stages of the analysis. One option is a solution in which the objective function is approximated by a non-linear regression, whereby the derivative of the new objective function should be polynomial, allowing the solution to the optimization problem to be calculated.

**Figure 4:**
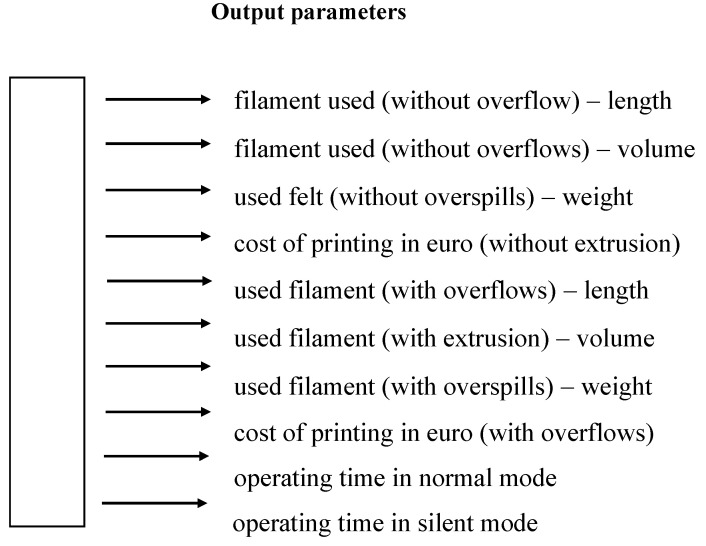
Output parameters of neural network.

The AI-based optimization included the following exoskeleton parameters (for a given part or group of parts, values expressed in SI units) (Table 1—input values, Figure 4—output values).

**Figure 5:**
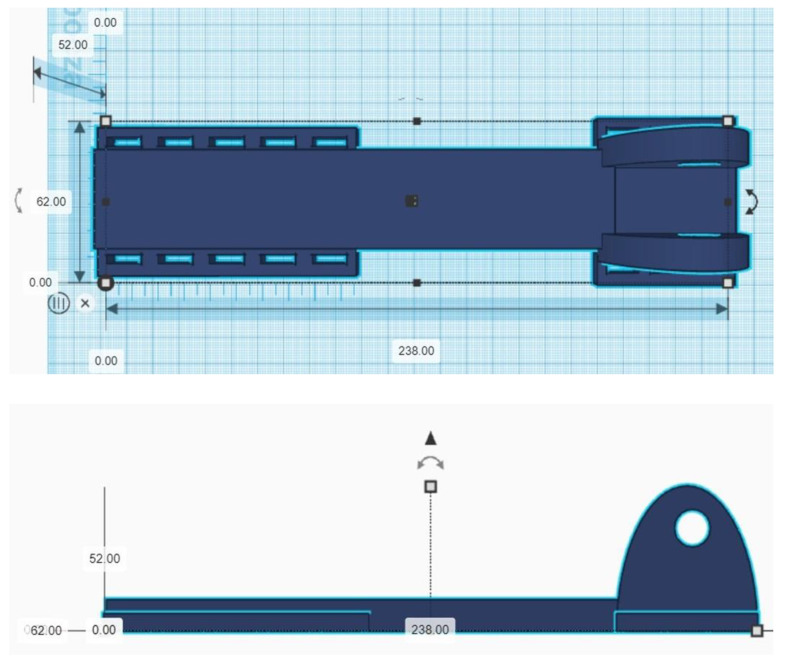
Optimized part of the exoskeleton.

The large number of input variables means that their full optimization is not always expedient or possible within a reasonable time frame applicable to industrial processes operating in real or near real-time. A network optimizing all print parameters should have an input layer of 238 and an output layer of 8 in dimension, which would require a network with about 438 neurons. The number of possible combinations is very large, but the actual technical capabilities of some of the parameters prevent certain combinations such as degrading the print quality, while in the study, we wanted to optimize the amount of waste without degrading the print quality (Figure 5).

MATLAB 16.0 (MathWorks, Portola Valley, USA) software including the Statistics and Machine Learning Toolbox was used to develop, test, and optimize the proposed ANN-based solution.

Datasets from research practice with 3D printers were used to learn the neural networks. The datasets were small, hence they were initially divided into three groups: learning (70% of the dataset), testing (20% of the dataset), and validation (10% of the dataset).

The input and output values were rescaled to values that fell within ranges with the same maximum and minimum values, and the initial values of the network weights were pre-estimated, normalized, and set to a range from −1 to 1. This prevented bias in the weights when the network was launched.

## 3. Results

**Figure 6:**
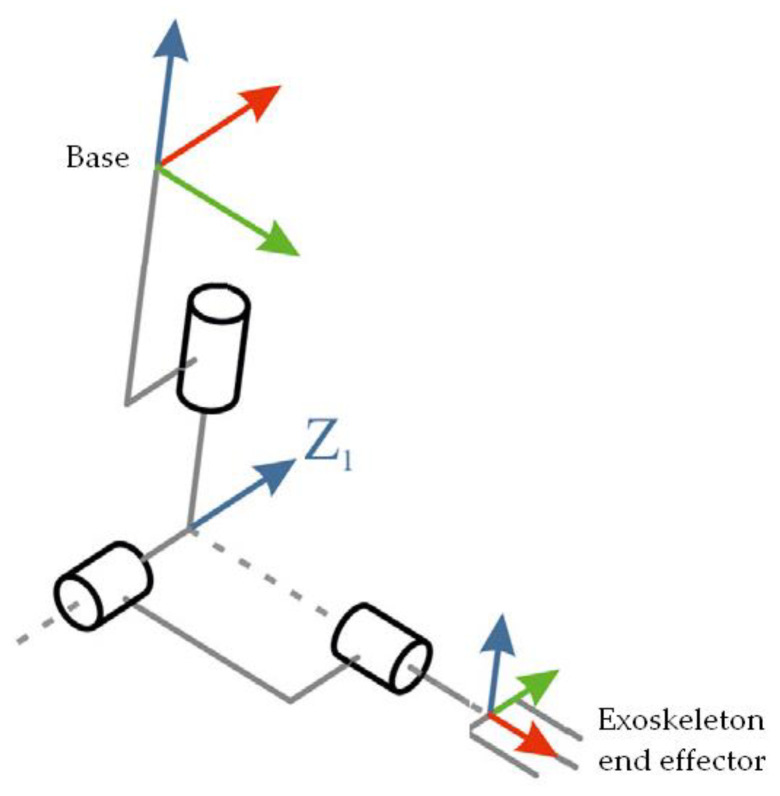
Kinematic structure of the elbow exoskeleton (based on [26]).

Concepts 1 and 2 presented in point 4 are based on the kinematic structure of the elbow exoskeleton shown in Figure 6. For the Z1 axis of rotation of the elbow (hinge joint), the arrow shows the axis of rotation. The vectors at the base show the vectors of the motions at the base. The joint/wrist moves freely in all directions without any loss in the range of these movements. The hand movements also support complete freedom of movement without any additional equipment. The cylinder mounts are marked with cylinders.

### 3.1. Concept 1 (External)

Using the above information, an upper limb model was constructed. The figure shows a discrete-concept model of the upper limb. A simplified model was used for the following reasons:

- Our thesis was to build and model an exoskeleton for the elbow; and
- The prediction of elbow movements was most important in this case and the rest of the joints (articulations) were less important.

**Figure 7:**
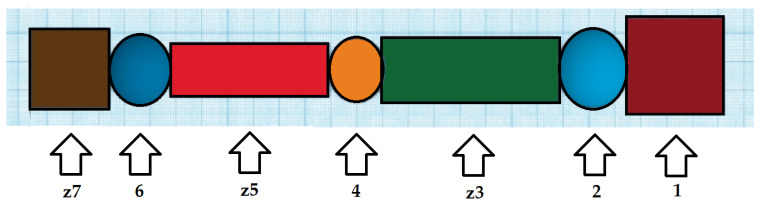
Exoskeleton project (1-Shoulder girdle; 2-scapular joint; z3-replacement arm; 4- elbow joint; z5-replacement forearm; 6 wrist joint; z7-replacement hand).

This was simplified to focus on the elbow movement. The hand was replaced with a single object (solid) rigid by virtue of not taking into account only the movement of the wrist (Figure 7).

The model meets specific criteria:

1. The form of the model:
  - Simplified model; and
  - Contains substitute elements not relevant for the modeling purpose (e.g., z7 simplified hand).
2. Model parameters:
  - Length and width of a given limb segment; and
  - The flexion angles of individual joints (hinges).
3. Conditions under which the model can be used—total biological accuracy.
4. Model applications is not important:
  - When modeling and constructing the ego-skeleton for the elbow/upper limb;
  - In the simulation of movements of the upper limb; and
  - In the manufacture of exoskeletons and digital devices that are designed to interact with the upper limb due to the discrete nature of the model.
5. The accuracy of the model that consists of simplified geometric figures.

**Figure 8:**
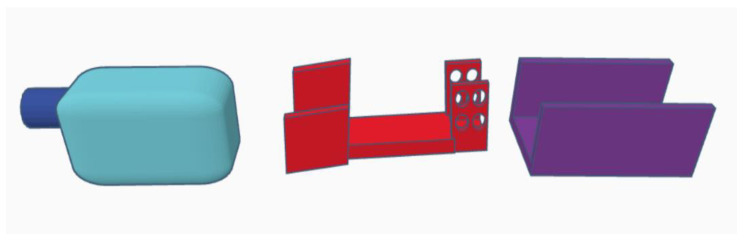
Parts of the exoskeleton: Concept 1 (external).

Parts of the exoskeleton (in Figure 8 from left): linear actuator, forearm attachment, and shoulder attachment. Each of the above parts is personalized to the user.

**Figure 9:**
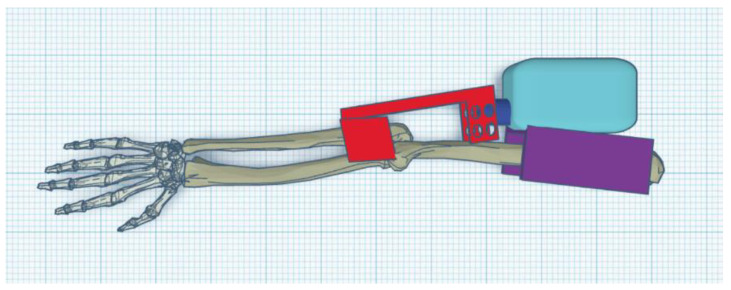
Upright exoskeleton with arm (0°).

**Figure 10:**
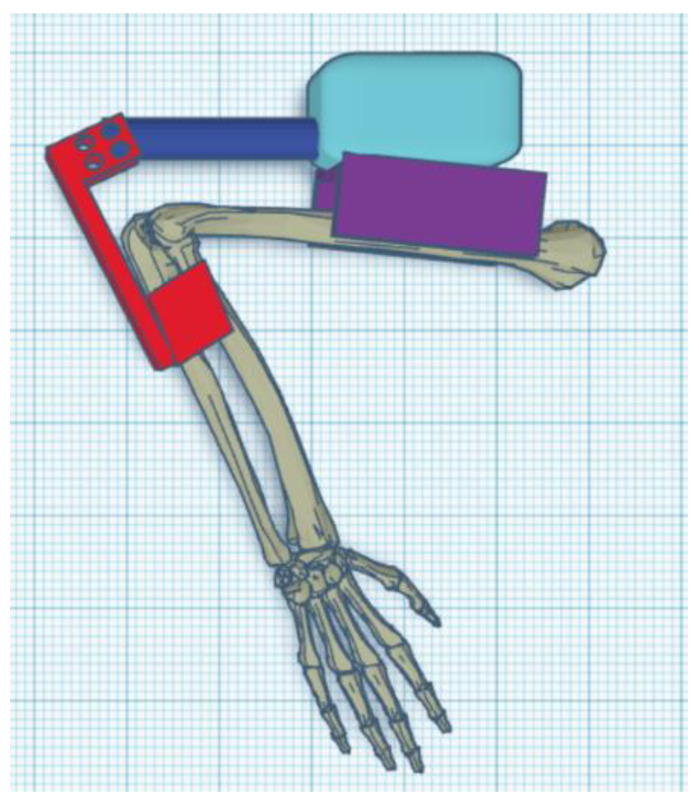
Flexed exoskeleton with arm (120°).

The biggest difference between the concept version and the prototype is getting rid of the large “wings”, which have been replaced by holders for the mounting straps. The reason for this was the need to give the muscles room to contract (Figure 8). The minimum angle of 90 degrees was a result of too-low positioning of the actuator in the arm section—the actuator should be moved toward the arm and lengthened to be able to mount the exoskeleton in the neutral position. The maximum angle obtained of 120 degrees is a physiological angle for humans. However, it should be noted that the forearm part of the exoskeleton has bent to about 10 degrees (Figure 9 and Figure 10).

### 3.2. Concept 2 (Internal)

**Figure 11:**
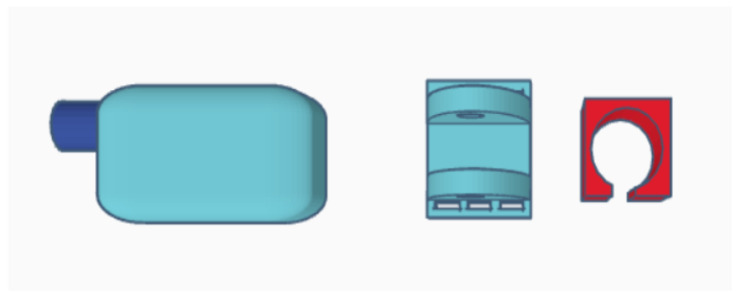
Parts of the exoskeleton: Concept 2 (internal).

Parts of the exoskeleton include (in Figure 11 from left): linear motor, forearm part, and shoulder part.

**Figure 12:**
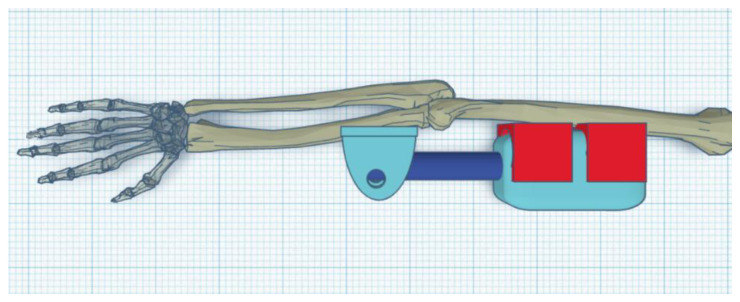
Upright exoskeleton with arm (0°).

**Figure 13:**
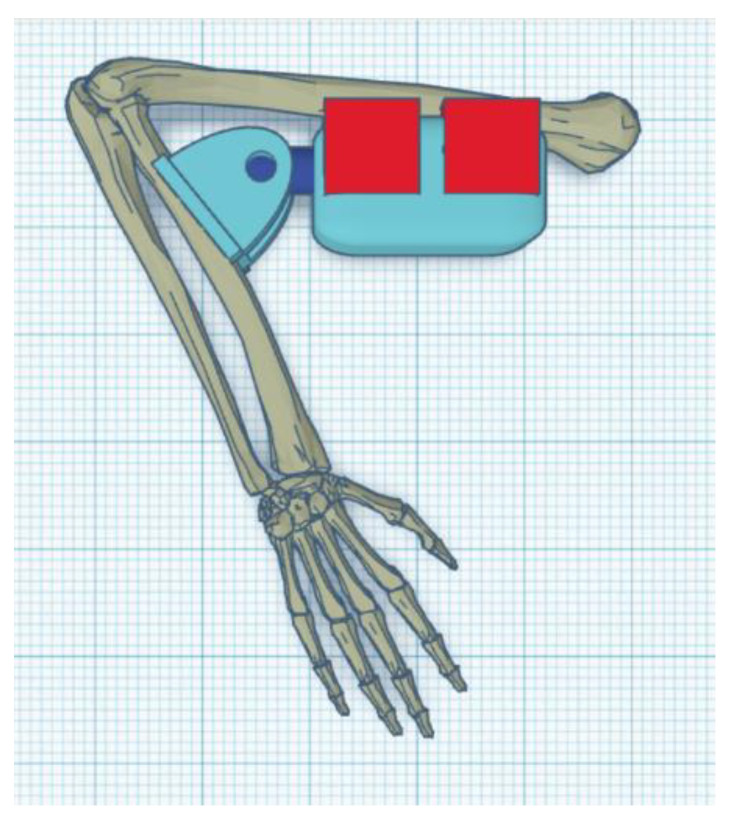
Flexed exoskeleton with arm (120°).

The exoskeleton is mounted on the humerus and forearm bone on the inside of the elbow (Figure 12 and Figure 13).

**Figure 14:**
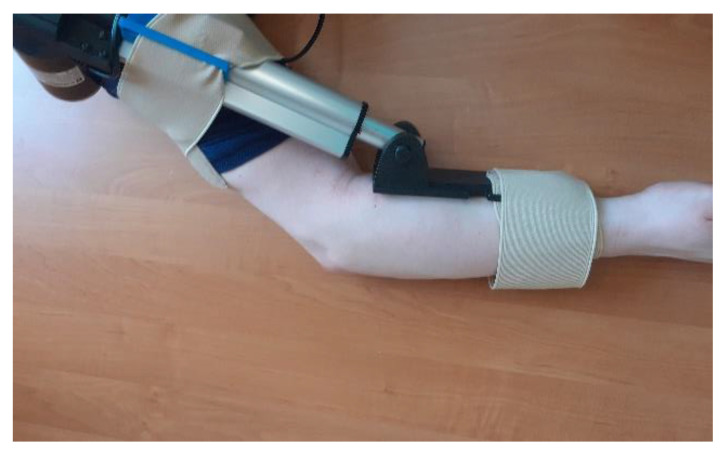
Exoskeleton on the user.

The minimum angle obtained of 20° was due to the dysfunctional elbow joint and its formation. The maximum angle was 90° (Figure 14).

The initial ANN was too complex for real-time use. While the learning process of the network can be done offline, the actual process of learning the ANN and calculating the parameters should be close to real-time, hence the importance of:

- Fast convergence;
- Simple ANN structure; and
- Small MSE.

After simplifying the network by reducing the number of input parameters, a simple ANN based on a multilayer perceptron can solve this problem.

Unfortunately, there is no automatic method to eliminate irrelevant input parameters. Networks with fewer parameters converge faster and result in lower error. Depending on the MLP network used, only the number of selected input parameters changed. A total of 238 parameters were selected as the input quantity (Table 1)—in this solution, the optimization does not take place in real-time. Accelerating the network to near real-time operation requires reducing the number of the above input parameters to 142 (Table 2). The removal of redundant parameters was done by the trial and error method accelerated considerably by the computational nature of the aforementioned study (i.e., by comparing the effects of removing successive input parameters). The area of optimization of neural network structures, especially the number of parameters important for the operation of the network in terms of its accuracy, but also the speed of minimization of MSE, is important for both industrial and biomedical systems and requires additional research.

**Figure 15:**
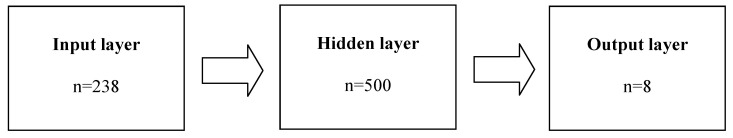
Optimal full-size ANN.

The original ANN MLP-238-500-8 (Figure 15) using sigmoid neurons was slow but achieved MSE for the data in the training set: 0.02, quality (learning): 0.8742, quality (testing): 0.9055.

**Figure 16:**
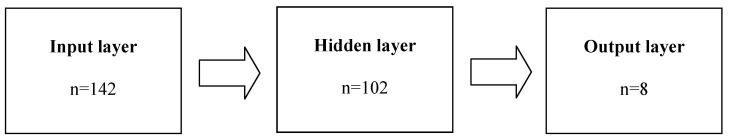
Optimal reduced-size ANN.

The ANN named MLP-142-102-8 (Figure 16) using sigmoid neurons was the best (after 1000 epochs): MSE for the data in the training set: 0.007, quality (learning): 0.8811, quality (testing): 0.9132 (Table 3).

We also tried to see whether the inclusion of less input data had an effect on the performance of the ANN. The ANN with the name MLP-50-52-8 achieved (after 1000 epochs):

- MSE for the data in the training set: 0.04;
- Quality (learning): 0.8977; and
- Quality (testing): 0.9283.

**Figure 17:**
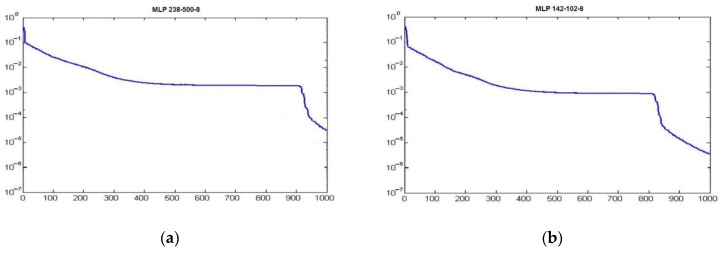
Values of mean square error (MSE) during learning: (**a**) MLP 238-500-8; (**b**) MLP 142-102-8).

**Figure 18:**
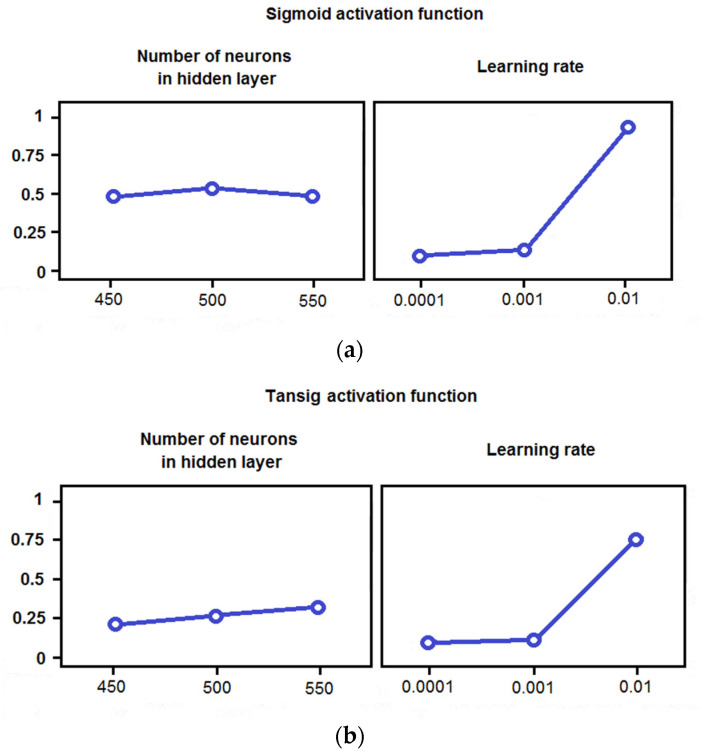
Main effects plots for MSE taking into account the activation function: (**a**) sigmoid function, (**b**) tansig function.

The calculation time was significantly shorter, but this is not a critical parameter in the present application—this is not a real-time system (Figure 17). It seems that the actual effects of the aforementioned optimization can be observed from the first moments of using the improved process regardless of the activation function (Figure 18).

The model before optimization weighed 0.07474 kg including an excess of 0.01124837 kg (i.e., 15.05%). After optimization, the model weighed 0.07268 kg including an excess of 0.0003634 kg (i.e., 0.05%). Thus, despite maintaining the print quality, the weight of the whole print decreased from 0.07474 kg to 0.7268 kg (i.e., by 0.00206 kg or by 2.7462%), and the waste weight itself decreased by 30.9531 times. This would allow for one free print after every 6.67 prints (i.e., from materials that were previously waste).

## 4. Discussion

Four main exoskeleton structures supporting the elbow joint have been identified thus far (Table 4).

The modeling of the exoskeleton structure based on the anatomy of the human upper limb, the personalized design of the exoskeleton based on human features from a 3D scan, and the functional study including movement at the elbow joint, the mechanical design, 3D printing as well as how to control, actuate, transfer power, and use and replace the various parts of the exoskeleton as they wear out have not been sufficiently covered to date. Not all problems have been effectively resolved yet, and technological advances may bring with them new optimal algorithms for analyzing biomedical signals and controlling the interaction of the exoskeleton with the user’s intention. Further research is needed to develop new mechanisms taking into account the complex biomechanics of the human elbow joint, especially for different types of diseases and injuries, causing different types and degrees of functional deficit and requiring different rehabilitation to support recovery [30].

The artificial intelligent capability of the system to generate new exercises, monitor the exercises performed by patients, evaluate progress, dynamically modify exercise characteristics, and troubleshoot becomes important as well as accurate sensors capable of collecting medical data allowing aggregation, inference, and prediction for greater accuracy in diagnosing, planning, and evaluating patient therapy.

AI-based software is useful, complementing existing design methods, and 3D printing software. Furthermore, more complex AI-based optimization solutions are needed, covering more steps in 3D printing processes. This could more significantly reduce air pollution, energy and material consumption, and provide a more optimal fit during 3D printing processes of medical devices such as the elbow exoskeleton presented here. An AI-based approach is required due to the need to adapt solutions to the Internet of Things and Industry 4.0 paradigms including the ability to technically monitor the entire production process, and respond to sensor signals and failures in real-time [31].

The main limitation of the study was the optimization of 3D printing only. A similar procedure should be applied comprehensively to both fabric parts, tendons, and electronic components. This would result in even greater savings including by recovering and reusing waste in the production process.

The execution time of computational tasks is not a critical parameter as there is no need to execute optimization tasks in real-time or near real-time on the production line. This is performed by a process engineer appropriately adjusting parameters of the production process and material.

The prices of plastics are increasing very quickly, as is their consumption, so it makes sense to optimize 3D printing procedures through, for example, improved design or injection simulation, allowing you to reduce the use of filament, save material, reduce waste, and reduce environmental impact. The cost-effectiveness of investing in the above-mentioned optimization software increases with the scale of production, and the investment pays for itself relatively quickly. The time and cost savings will not reduce the high quality of the products, and can provide a competitive advantage, especially in the case of mass-produced products designed to be very thin.

It should be noted that the above-mentioned utilization (closed loop) should, as far as possible, take place within the same production line, so as not to add additional transport needs, etc., nullifying the effort of waste reduction and reuse. It seems that both the optimization of the use of materials itself and its widespread application should become obligatory practice. However, this requires not only public and business awareness, but also legal regulations as part of a sensible strategy for entire countries, and in the initial period of use as well as incentives in the form of tax breaks and building an image of resource-efficient companies [32,33,34].

The directions for further studies include computational optimization of datasets using a broader spectrum of AI methods such as directed fuzzy numbers and fractal parameters.

## 5. Conclusions

Elbow exoskeleton systems are important in assisting movement and rehabilitation. The physical interaction between the human body parts and the exoskeleton is very important and has not been satisfactorily addressed in the most recently developed systems for the elbow joint.

The device presented in this paper is a mechatronic system with features of a wearable robot reproducing the kinematic structure of the human upper limb creating a parallel kinematic chain: two degrees of freedom with two active elements.

The parameters of the flexible element were determined on the basis of a dynamic model of the elbow joint and data on human perceptual limitations. The device and its control system were developed to interact with the human body with force feedback interaction of 400 N.

The original ANN MLP-238-500-8 was slow, but achieved MSE for the data in the training set: 0.02, quality (learning): 0.8742, and quality (testing): 0.9055. MLP-142-102-8 using sigmoid neurons was the best (after 1000 epochs) achieving MSE for the data in the training set: 0.007, quality (learning): 0.8811, and quality (testing): 0.9132.

AI-based optimization can improve the environmental friendliness of the exoskeleton without compromising its quality. This would allow for one free print after every 6.67 prints (i.e., from materials that were previously waste).

## Figures and Tables

**Table 1:** Input parameters of the neural network.

Fixed Input Parameters	
0.4 mm nozzle width (these nozzles represent about 60% of the market), printed part and its spatial characteristics (in order to unify requirements)	
Variable input parameters	
Printer Setting	Avoid crossing: perimeters, perimeters max detour Bed: custom model, custom texture, shape, temperature, before layer gcode, between objects gcode Bottom: fill pattern, solid layers, solid min thickness, bridge acceleration, bridge angle, bridge fan speed, bridge flow ratio, bridge speed, brim width, clip multipart objects, color change gcode, compatible printers condition cumulative, complete objects Cooling: tube length, tube retraction, default acceleration, default filament profile, default print profile, deretract speed, disable fan first layers, don’t support bridges, draft shield, duplicate distance, elephant’s foot compensation, end filament gcode, end gcod, ensure vertical shell thickness External perimeter: extrusion width, speed, first, move, extra perimeters Extruder: clearance height, clearance radius, color, offset, axis, multiplier, width, fan always on, fan below layer time
Filament Setting	Filament: color, cooling final speed, cooling initial speed, cooling moves, cost, density, diameter, load time, loading speed, loading speed start, max volumetric speed, minimal purge on wipe tower, notes, ramming parameters, settings id, soluble, spool weight, tool change delay, type, unload time, unloading speed, unloading speed start, vendor Fill: angle, density, pattern First layer: acceleration, bed temperature, extrusion width, height, speed, temperature, full fan speed layer, gap fill speed, gcode comments, gcode flavor, gcode label objects, high current on filament swap, host type Infill: acceleration, anchor, anchor max, every layers, extruder, extrusion width, first, only where needed, overlap, speed, interface shells Ironing: flowrate, spacing, speed, type, layer gcode, layer height Machine: limits usage, max acceleration e, max acceleration extruding, max acceleration retracting, max acceleration x, max acceleration y, max acceleration z, max feedrate e, max feedrate x, max feedrate y, max feedrate z, max jerk e, max jerk x, max jerk y, max jerk z, min extruding rate, min travel rate, max fan speed Layers and perimeters: max layer height, max print height, max print speed, max volumetric speed, min fan speed, min layer height, min print speed, min skirt length, notes, nozzle diameter, only retract when crossing perimeters, ooze prevention, output filename format, overhangs, parking pos retraction, pause print gcode, perimeter acceleration, perimeter extruder, perimeter extrusion width, perimeter speed, perimeters, physical printer settings id, post process Printer: print settings id, model, notes, settings id, technology, variant, vendor, raft layers, remaining times, resolution Retract: before travel, before wipe, layer change, length, length tool change, lift, lift above, lift below, restart extra, restart extra tool change, speed, seam position, silent mode, single extruder multi material, single extruder multi material priming, skirt distance, skirt height, skirts, slice closing radius, slowdown below layer time, small perimeter speed Solid infill: below area, every layers, extruder, extrusion width, speed
Support Setting	Spiral vase: vase, standby temperature delta, start filament gcode, start gcode Support material: angle, auto, build plate only, contact distance, enforce layers, extruder, extrusion width, interface contact loops, interface extruder, interface layers, interface spacing, interface speed, pattern, spacing, speed, synchronize layers, threshold, with sheath, xy spacing, temperature, template custom gcode, thin walls
Other Setting	Threads: thumbnails, tool change gcode, top fill pattern, top infill extrusion width, top solid infill speed, top solid layers, top solid min thickness, travel speed, use firmware retraction, use relative distances, use volumetric e, variable layer height Wipe: into infill, into objects, tower, tower bridging, tower no sparse layers, tower rotation angle, tower width, tower x, tower y, wiping volumes extruders, wiping volumes matrix, xy size compensation, z offset

**Table 2:** Decreased number of 142 input parameters of the neural network.

Fixed Input Parameters	
0.4 mm nozzle width (these nozzles represent about 60% of the market), printed part and its spatial characteristics (in order to unify requirements)	
Variable input parameters	
Printer Setting	Avoid crossing: perimeters, perimeters max detour Bed: custom model, custom texture, shape, temperature, before layer gcode, between objects gcode Bottom: fill pattern, solid layers, solid min thickness, bridge acceleration, bridge angle, bridge fan speed, bridge flow rati, bridge speed, brim width, clip multipart objects, color change gcode, compatible printers condition cumulative, complete objects Cooling: tube length, tube retraction, default acceleration, default filament profile, default print profile, deretract speed, disable fan first layers, don’t support bridges, draft shield, duplicate distance, elefant foot compensation, end filament gcode, end gcod, ensure vertical shell thickness External perimeter: extrusion width, speed, first, move Extruder: clearance height, clearance radius, color, offset, axis, multiplier, width, fan always on, fan below layer time
Filament Setting	Filament: color, cooling final speed, cooling initial speed, cooling moves, density, diameter, loading speed start, minimal purge on wipe tower, ramming parameters, soluble, spool weight, tool change delay Fill: angle, density, pattern First layer: acceleration, bed temperature, extrusion width, height, speed, temperature, full fan speed layer, gap fill speed, gcode label objects, high current on filament swap, Infill: acceleration, anchor, extrusion width, first, only where needed, overlap, speed Ironing: flowrate, spacing, speed, layer height Layers and perimeters: nozzle diameter, only retract when crossing perimeters, overhangs, perimeter acceleration, perimeter extruder, perimeter extrusion width, perimeter speed, perimeters, post process Printer: print settings id, settings id, technology, variant, raft layers, remaining times, resolution Retract: before travel, before wipe, layer change, length, length tool change, lift, lift above, lift below, restart extra, restart extra tool change, speed, seam position, silent mode, single extruder multi material, single extruder multi material priming, skirt distance, skirt height, skirts, slice closing radius, slowdown below layer time, small perimeter speed Solid infill: below area, every layers, extruder, extrusion width, speed
Support Setting	Spiral vase: vase, standby temperature delta, start filament gcode, start gcode Support material: angle, auto, build plate only, contact distance, enforce layers, extruder, extrusion width, interface contact loops, interface extruder, interface layers, interface spacing, interface speed, pattern, spacing, speed, synchronize layers, threshold, with sheath, xy spacing, temperature, template custom gcode, thin walls

**Table 3:** Selected ANN quality assessment and MSE values (bold shows the best result).

Network Name	Quality (Learning)	Quality (Testing)	MSE
MLP 142-80-8	0.8723	0.9022	0.01
MLP 142-102-8	0.8811	0.9132	0.007
MLP 142-136-8	0.8712	0.9101	0.02
MLP 142-160-8	0.8743	0.9118	0.02

**Table 4:** The most advanced exoskeleton structures supporting the elbow joint.

Name	Research Team	Number of Joints	Type and Location of Drive Units
ExoArm 7-DOF	West Pomeranian University of Technology, Szczecin, Poland [26]	7 active	Closed-loop Bowden cable conduit system
EXO-UL8	Bionics Lab, University of California Los Angeles, USA [27]	8 active	Dynamometric actuators located directly on the device structure
IntelliArm	Rehabilitation Institute of Chicago, USA [28]	7 active, 2 passive	Actuators placed directly at the joints
SUEFUL-7	Saga University, Japan [29]	7 active	DC motors directly drive 4 joints, 3 joints are driven by cable transmission

## Data Availability

Data sharing is not applicable to this article.
